# Supplementary material for: Patterns and predictors of variability in patient-generated daily pain severity collected via a mobile health smartphone app
Source: PLoS One. 2026 Apr 2;21(4):e0345420. doi: 10.1371/journal.pone.0345420 (PMC13046152; doi:10.1371/journal.pone.0345420)
Supplement: Attachment — Submitted filename: Response to Reviewers.docx [file pone.0345420.s002.docx]

**PONE-D-25-45216**

**Patterns and predictors of variability in patient-generated daily pain severity collected via a mobile health smartphone app**

***PLOS One***

Author responses are in red.

**RESPONSE TO JOURNAL REQUIREMENTS:**

When submitting your revision, we need you to address these additional requirements.

1. Please ensure that your manuscript meets PLOS ONE's style requirements, including those for file naming. The PLOS ONE style templates can be found at

[https://journals.plos.org/plosone/s/file?id=wjVg/PLOSOne_formatting_sample_main_body.pdf [journals.plos.org]](https://urldefense.com/v3/__https:/journals.plos.org/plosone/s/file?id=wjVg*PLOSOne_formatting_sample_main_body.pdf__;Lw!!PDiH4ENfjr2_Jw!DhTmqJC0iFROPrGst6lGhN9QHCIqta-XszUHGwqpRkeLc5uLGLq8hw3zsx4ejfhPMlH6Dxmgvt7KuDmimEL50nB1n5I$) and [https://journals.plos.org/plosone/s/file?id=ba62/PLOSOne_formatting_sample_title_authors_affiliations.pdf [track.editorialmanager.com]](https://urldefense.com/v3/__https:/track.editorialmanager.com/CL0/https:*2F*2Fjournals.plos.org*2Fplosone*2Fs*2Ffile*3Fid=ba62*2FPLOSOne_formatting_sample_title_authors_affiliations.pdf/1/010f019b01a633a6-1b46fc7d-7d96-4b74-9b06-8435795c26e2-000000/_Y3f0a56GKW1lT8VSw7mpHRNrEIEAeWhYTYBjSNYdB4=240__;JSUlJSUlJQ!!PDiH4ENfjr2_Jw!DhTmqJC0iFROPrGst6lGhN9QHCIqta-XszUHGwqpRkeLc5uLGLq8hw3zsx4ejfhPMlH6Dxmgvt7KuDmimEL5BGw6QPI$)

The title page and manuscript have now been revised in accordance with these documents.

2. We note that the grant information you provided in the ‘Funding Information’ and ‘Financial Disclosure’ sections do not match.

When you resubmit, please ensure that you provide the correct details and grant numbers for the awards you received for your study in the ‘Funding Information’ section.

These two sections are now the same with the correct details and grant numbers.

The revised Funding Statement can be found in the new cover letter.

3. Thank you for stating in your Funding Statement:

[This study was supported by the Centre for Epidemiology Versus Arthritis (grant number 21755). Cloudy with a Chance of Pain was funded by Versus Arthritis (grant reference 21225), with additional support from the Centre for Epidemiology (grants 21755 and 20380). TH receives funding from the Royal Society (grant number INF/R2/180067) and the Alan Turing Institute for Data Science and Artificial Intelligence.].

Please provide an amended statement that declares *all* the funding or sources of support (whether external or internal to your organization) received during this study, as detailed online in our guide for authors at [http://journals.plos.org/plosone/s/submit-now [journals.plos.org]](https://urldefense.com/v3/__http:/journals.plos.org/plosone/s/submit-now__;!!PDiH4ENfjr2_Jw!DhTmqJC0iFROPrGst6lGhN9QHCIqta-XszUHGwqpRkeLc5uLGLq8hw3zsx4ejfhPMlH6Dxmgvt7KuDmimEL53roSRCw$).  Please also include the statement “There was no additional external funding received for this study.” in your updated Funding Statement.

Please include your amended Funding Statement within your cover letter. We will change the online submission form on your behalf.

The revised Funding Statement can be found in the new cover letter.

4. Thank you for stating the following in the Acknowledgments Section of your manuscript:

[This study was supported by the Centre for Epidemiology Versus Arthritis (grant number 21755). Cloudy with a Chance of Pain was funded by Versus Arthritis (grant reference 21225), with additional support from the Centre for Epidemiology (grants 21755 and 20380). Ethical approval for Cloudy with a Chance of Pain was from the University of Manchester Research Ethics Committee (ref: ethics/15522) and the NHS IRAS (ref: 23/NW/0716). TH receives funding from the Royal Society (grant number INF/R2/180067) and the Alan Turing Institute for Data Science and Artificial Intelligence. The funders of the study had no role in study design, data interpretation, writing of the manuscript, or the decision to submit for publication.]

We note that you have provided funding information that is currently declared in your Funding Statement. However, funding information should not appear in the Acknowledgments section or other areas of your manuscript. We will only publish funding information present in the Funding Statement section of the online submission form.

Please remove any funding-related text from the manuscript and let us know how you would like to update your Funding Statement. Currently, your Funding Statement reads as follows:

[This study was supported by the Centre for Epidemiology Versus Arthritis (grant number 21755). Cloudy with a Chance of Pain was funded by Versus Arthritis (grant reference 21225), with additional support from the Centre for Epidemiology (grants 21755 and 20380). TH receives funding from the Royal Society (grant number INF/R2/180067) and the Alan Turing Institute for Data Science and Artificial Intelligence.]

Please include your amended statements within your cover letter; we will change the online submission form on your behalf.

We have deleted funding-related text from the manuscript. The Funding Statement has been revised in accordance with your comment 3. It can be found in the new cover letter.

5. Thank you for stating the following in the Competing Interests section:

[I have read the journal's policy and the authors of this manuscript have the following competing interests: WGD has received consultancy fees from Google, and DMS has received consultancy fees from Palta, both unrelated to this work. All other authors have declared that no competing interests exist.].

Please confirm that this does not alter your adherence to all PLOS ONE policies on sharing data and materials, by including the following statement: "This does not alter our adherence to PLOS ONE policies on sharing data and materials.” (as detailed online in our guide for authors [http://journals.plos.org/plosone/s/competing-interests [journals.plos.org]](https://urldefense.com/v3/__http:/journals.plos.org/plosone/s/competing-interests__;!!PDiH4ENfjr2_Jw!DhTmqJC0iFROPrGst6lGhN9QHCIqta-XszUHGwqpRkeLc5uLGLq8hw3zsx4ejfhPMlH6Dxmgvt7KuDmimEL5FBeHV3c$)).  If there are restrictions on sharing of data and/or materials, please state these. Please note that we cannot proceed with consideration of your article until this information has been declared.

We have added the statement “This does not alter our adherence to PLOS ONE policies on sharing data and materials.” to the Declaration of Interests statement.

Please include your updated Competing Interests statement in your cover letter; we will change the online submission form on your behalf.

6. You have indicated that data is available from [[elaine.mackey@manchester.ac.uk](mailto:elaine.mackey@manchester.ac.uk)].  Please can we ask you to provide us with a general contact email address for the data requests, so readers can request access in perpetuity. If a general email is not available please provide a link to a website where readers can obtain access to data.

Because of the complexity of anonymizing our data and making it available in a way that we can share, we are trying to make the dataset available through Figshare. However, this route is proving more complex than we hoped. We are working towards a solution, but it is going to take some time (probably mid-year).  As the review process is seemingly to continue, may we be allowed to proceed? We are working on a solution. In the meantime, please can they use the contact previously given (or [will.dixon@manchester.ac.uk](mailto:will.dixon@manchester.ac.uk), the PI of the project). Once we have a solution in place, we can update you.

7. Your ethics statement should only appear in the Methods section of your manuscript. If your ethics statement is written in any section besides the Methods, please move it to the Methods section and delete it from any other section. Please ensure that your ethics statement is included in your manuscript, as the ethics statement entered into the online submission form will not be published alongside your manuscript.

The ethics statement now appears only in the methods section (second paragraph of section 2). We have deleted it from the acknowledgements.

If the reviewer comments include a recommendation to cite specific previously published works, please review and evaluate these publications to determine whether they are relevant and should be cited. There is no requirement to cite these works unless the editor has indicated otherwise.

None of the reviewers have requested citation to previously published works.

**RESPONSE TO REVIEWERS’ COMMENTS:**

Reviewer's Responses to Questions

Thank you to the two reviewers for their positive feedback on the manuscript. Their suggestions have transformed the manuscript considerably into a much more readable piece of work. We appreciate their time and effort reading our manuscript and providing their comments.

Comments to the Author

1. Is the manuscript technically sound, and do the data support the conclusions?

The manuscript must describe a technically sound piece of scientific research with data that supports the conclusions. Experiments must have been conducted rigorously, with appropriate controls, replication, and sample sizes. The conclusions must be drawn appropriately based on the data presented.

Reviewer #1: Yes

Reviewer #2: Yes

2. Has the statistical analysis been performed appropriately and rigorously?

Reviewer #1: I Don't Know

Reviewer #2: Yes

3. Have the authors made all data underlying the findings in their manuscript fully available?

The PLOS Data policy requires authors to make all data underlying the findings described in their manuscript fully available without restriction, with rare exception (please refer to the Data Availability Statement in the manuscript PDF file). The data should be provided as part of the manuscript or its supporting information, or deposited to a public repository. For example, in addition to summary statistics, the data points behind means, medians and variance measures should be available. If there are restrictions on publicly sharing data—e.g. participant privacy or use of data from a third party—those must be specified.

Reviewer #1: Yes

Reviewer #2: Yes

4. Is the manuscript presented in an intelligible fashion and written in standard English?

PLOS ONE does not copyedit accepted manuscripts, so the language in submitted articles must be clear, correct, and unambiguous. Any typographical or grammatical errors should be corrected at revision, so please note any specific errors here.

Reviewer #1: Yes

Reviewer #2: Yes

5. Review Comments to the Author

Please use the space provided to explain your answers to the questions above. You may also include additional comments for the author, including concerns about dual publication, research ethics, or publication ethics. (Please upload your review as an attachment if it exceeds 20,000 characters)

**RESPONSE TO REVIEWER 1:**

Reviewer #1: Review of PONE-D-25-45216:

Patterns and predictors of variability in patient-generated daily pain severity collected via a mobile health smartphone app (Schultz et al.)

Overview:

This manuscript addresses an important question: how daily pain variability can be characterized using high-frequency patient-generated data from a large mHealth cohort. The dataset is rich and the basic scientific-clinical question is solid. The work builds on the prior “Cloudy with a Chance of Pain” studies and extends them meaningfully by focusing specifically on variability. That said, this paper has some methodological and mostly reporting flaws that warrant correction before publication.

To Reviewer 1, thank you for your kind words on our manuscript. You have identified a number of instances where we could have been more clear or understandable. We hope that our revised manuscript addresses the flaws that you identified.

Major Comments:

1. This paper is extremely technical. As a physician with only moderate understanding of statistics I found it very hard to understand. Moreover, while I have faith in the authors that it is is mathematically correct (too technical for me to review), I could not make out the narrative of the paper, i.e, there is simple “big picture” section guiding the reader through the logic of the analysis before the numbers appear. Key technical terms (AC, PAC, MSSD) are not well explained for the non-expert readers. Understanding these is key to understanding the paper entirely.

Writing a journal article that speaks to a variety of different readers with different expertises and technical knowledge is not easy. *PLOS One* offers us authors an opportunity to get our work exposed to such a variety of different readers, but we understand that perhaps our manuscript did not meet the mark in your case.

Naturally, some readers will be interested in the technical details, and we should include as much of those technical details as is needed for them to understand our methods and their interpretation, so that they can reproduce our work on their own.

Nevertheless, we are sympathetic to the reviewer’s concerns. We want to make our manuscript more easily accessible to all potential readers, so we appreciate your efforts to call out opportunities for us to do so. Therefore, we have made the following revisions to address your concerns.

1. To make the manuscript easier to understand with a clearer narrative, we have tried to explain our approach also in plain language. Looks for revisions scattered throughout the Data and Methods section and the Results section. We have removed acronyms where possible, such as PGHD, BIC, and mHealth.

2. For the Data and Methods section, we have added an introductory paragraph that explains the structure of this section, providing the “big picture” that the reviewer requests.

3. The Data and Methods section is then broken down into four subsections that have descriptive titles that reuse the same language from the introductory paragraph, further justifying and clarifying the data and the steps it was subjected to for processing.

- Collecting data: The Cloudy with a Chance of Pain study
- Constructing weekly pain-trajectory data
- Constructing weekly clusters of pain trajectories and quantifying day-to-day variability
- Identifying variables associated with day-to-day variability in pain severity

4. The Results section has two subsections with similar descriptive titles, helping signpost the reader.

- Quantifying day-to-day variability in pain severity among the four clusters
- Variables associated with day-to-day variability in pain severity among the four clusters

5. The Results section has more text to help explain to the reader the reasons for the analysis, figures, and tables. Paragraphs and sentences have been rearranged to hopefully make a better flow in some places.

6. Both the Discussion and Conclusion section have been restructured for focus, clarity, and content. The Conclusion section now comprises a longer summary of our results, including in more plain language for easier reading.

We hope you find these revisions satisfactory.

2. Similarly, I feel like the paper focuses too much on mathematical findings and too little on patient-centered outcomes. It jumps between terms like stable weeks, variability metrics, LASSO-selected predictors and without interperting these into outcomes that matter to patients. This also menifests in the fact the discussion is very short compared to other parts of the manuscript.

We have revised the balance of content between the Discussion and the Conclusion sections, adding content that addresses the reviewer’s comment #5. The revised Discussion section focuses on comparing our results to previous literature, discussing the strengths and weaknesses of the study, the importance of this research to patient-centered outcomes, and potential future research.

Although we are highly motivated to improve participant understanding of their pain, that was not the purpose of this manuscript. We cannot make the technical aspects of this work go away, as they are essential for the calculations for understanding the variability of pain and its associations. Much more work would be needed for patient-centered outcomes, probably involving a patient and public involvement focus group. As such, this further research has not been done and would be out of scope of the present manuscript.

Nevertheless, we are sympathetic to the reviewer’s concern. We have added more explanation of our results with a focus on patient-centered outcomes to the Discussion section.

3. The Methods section is in my view overly long and somewhat cumbersome. Honestly, although reading it twice, I am not sure I understood the Methods correctly. It includes many internal definitions (e.g. available weeks, covariate score), the reasoning behing choosing different options in the methods (e.g. why a week starts on Monday), very in-depth statistical data, and goes into so much detail about the methodology that for the reader unfamiliar with the previous work, it becomes impossible to understand. I recommend revising the Methods to make it much shorter – e.g. one parageaph for each of the subsections. I also recommend adding an explanatory figure. If the authors still want to include what’s currently described in the Methods, I recommend adding a supplementary appendix.

As we discuss earlier in our response, we are writing this manuscript for multiple audiences. Deleting content from the Data and Methods section would be inappropriate for the subset of readers interested in the technical details of our methods who would want to replicate our methods and analysis on their own datasets.

We examined different studies on pain trajectories previously published in *PLOS One*, and we don’t see any evidence that authors had placed detailed descriptions of methods into a supplement or appendix. Moreover, doing so would require us to explain our methods twice, once cursorily in the body of the manuscript and once in more detail in the supplement/appendix.

As such, we believe it is appropriate to include more big-picture language and signposting to help guide readers through the Data and Methods section. These revisions manifest most apparently in two ways: an introductory paragraph and restructuring the section into four subsections.

First, we have added an introductory paragraph at the beginning of this section that provides an overview of the section and the various steps from data collection to data processing to data analysis. We believe that this paragraph clearly explains our data and methods sufficiently and accomplishes that more effectively than would an explanatory figure.

Second, the Data and Methods section is broken up into four subsections with more descriptive titles in a natural language that mirror the language used in this introductory paragraph.

- Collecting data: The Cloudy with a Chance of Pain study
- Constructing weekly pain-trajectory data
- Constructing weekly clusters of pain trajectories and quantifying day-to-day variability
- Identifying variables associated with day-to-day variability in pain severity

Other changes that we have undertaken to improve the readability of the Data and Methods section include rearranging text into the four subsections for a more smoother flow, reducing redundancies of text, and adding more plain-language description of the methods to help signpost the readers through this content.

Along with improved explanation for the technical details in natural language in addition to the more technical language already existing, we hope that these revisions help improve the readability of our manuscript for you, without sacrificing the details necessary for other readers who require more technical detail to replicate the study design and methods.

4. All tables and figures require legends (which include explanation of acronyms), and should be referenced from and support the arguments made in the main text. Currently many just report a lot of data. Similarly I feel that some data reported in the supplement (e.g. demographics) should be transferred to the main manuscript file, and vice versa.

It is unclear what the reviewer means by all tables and figures require legends. All in the original manuscript had captions, and those that needed legends had them embedded within the figure. All figures and tables were cited within the text in the original manuscript.

The demographics table has been moved to the manuscript (new Table 1).

As the reviewer did not state which figures should be moved to the supplement, we leave them in the manuscript. All figures now reside in the manuscript, which we think improves the readability and accessibility of the manuscript for all readers.

Finally, we aimed to provide more discussion of the results of each figure and table in the revised manuscript. Look especially at the discussions in the manuscript for Fig 2 and Table 6.

We hope these revisions improve the manuscript.

5. This study includes several limitations that should either be mitigated, better reported, or at the very least mentioned in the Discussion (which oddly does not include a “limitations” section). These include:

A. substantial loss of weeks due to incomplete covariate data

B. Demographics are highly skewed toward females and toward younger patients.

C. It is unclear whether complete weeks differ systematically in key outcomes (pain levels, variability) compared to incomplete weeks.

A new paragraph is added to the Discussion section to discuss these limitations (A and C have been combined into one).

6. Conclusions section is very long and includes parts that don’t belong in it. Conclusions should be one paragraph summarizing the paper, everything else belongs in the general Discussion.

This request is inconsistent with the guidance to *PLOS One* authors (<https://journals.plos.org/plosone/s/submission-guidelines>). Specifically, the guidance is quite flexible for how authors choose to write Discussion and Conclusion sections. Moreover, that guidance does not indicate that the Conclusion should be limited to one paragraph.

Nevertheless, the reviewer has a good point. We agree that the Conclusion should focus on summarizing the results of the paper. Our revised Conclusion section does that by moving the last two paragraphs pertaining to the strengths and future work to the Discussion section, where we believe these paragraphs are more appropriate.

We disagree, however, with the recommendation to make the Conclusion one paragraph. If the Conclusion were only one paragraph long, it would largely replicate the abstract, thereby being unnecessary and redundant. We argue that the Conclusion section should summarize the paper with more substance and depth than the abstract. The revised Conclusion is four paragraphs long and about 400 words. This is concise, but also with more depth than our abstract.

We hope you agree that the revised Discussion and Conclusion sections are satisfactory.

Minor Comments:

1. Abstract mentions four clusters but does not explain clustering methodology. Suggest including a short explanation as exists in the full paper (e.g. no, mild, moderate and severe pain). In general, for the sake of simplicity I recommend using the actual term (mild-moderate-severe) instead of the A-B-C-D labeling which may add confusion.

Two good points that we are happy to adopt. We have renamed the clusters as you suggest. Also, the abstract has now been revised to include the following text.

“Summary statistics and distributions of pain changes on consecutive days were calculated within 13,052 complete weeks of data, which had been assigned to one of four clusters via a previously published k-mediods clustering algorithm: no/low pain, mild pain, moderate pain, and severe pain.”

2. I find it odd that two figures in the paper describe a hypothetical patient. Figures should depict data collected.

Only one figure (former Fig 1, present Fig 2) is for a hypothetical participant. The other figure (former Fig 2, present Fig 1) shows actual data, as per the figure caption: “Example trajectory weeks, taken from data previously assigned to Cluster C (“moderate pain”).”

It is unclear where the rule can be found that only actual data needs to be depicted in figures in a manuscript (<https://journals.plos.org/plosone/s/figures>). We have seen many other scientific manuscripts where hypothetical or theoretical examples were used.

In the former Fig 1, it was easier and more illustrative to construct a time series for a hypothetical participant than to search for actual data in our dataset that had all the requisite features we wished to show. As such, we prefer to keep the hypothetical data as it is.

We have made no change to the manuscript in response to this comment.

3. References: Schneider et al. (2012) is duplicated.

One of the references to Schneider et al. (2012) has been deleted. Thank you.

4. Figure 3b (graphical distribution of pain differences) is informative but difficult to interpret without a color scale or more explicit labeling.

This figure panel replicates the information in the former Fig 3a (present Fig 3). As you feel that the former Fig 3b is not easy to interpret and we believe it is redundant, we have deleted this figure panel in the revised manuscript.

5. Note some minor typos throughout the manuscript (e.g. inteference).

“Interference” has been fixed. We have changed British English to American English throughout because *PLOS One* is an American journal. We have looked for other typos but have found few, if any. We have fixed them where we have encountered them.

**RESPONSE TO REVIEWER 2:**

Reviewer #2: I congratulate you for your detailed analysis and meticulously prepared research, as well as for your clearly written article. However, there are some points that I would like to draw attention to.

Thank you for your kind words. Your comments have improved our manuscript.

-What does "pain interfence" mean, which you mentioned on page 11, lines 358-359? Could you explain this a little more?

This was just a typo for “interference” that was unfortunately replicated across several locations. We have fixed this in the revised manuscript.

- The conclusion section is longer than the discussion section. Shouldn't the information in the conclusion section be included in the discussion section?

The guidance to *PLOS One* authors for Discussion and Conclusion sections is quite flexible (<https://journals.plos.org/plosone/s/submission-guidelines>). Nevertheless, we agree that the structure of these two sections in our original submission could have been improved to more clearly distinguish between discussion-type material for the Discussion and the summary of the manuscript for the Conclusion. We hope you agree that the revised manuscript is better structured.
