# Supplementary material for: Patterns and predictors of variability in patient-generated daily pain severity collected via a mobile health smartphone app
Source: PLoS One. 2026 Apr 2;21(4):e0345420. doi: 10.1371/journal.pone.0345420 (PMC13046152; doi:10.1371/journal.pone.0345420)
Supplement: Attachment — Submitted filename: Response_to_Reviewers_auresp_2.docx [file pone.0345420.s003.docx]

**PONE-D-25-45216**

**Patterns and predictors of variability in patient-generated daily pain severity collected via a mobile health smartphone app**

***PLOS One***

Author responses are in red.

**RESPONSE TO JOURNAL REQUIREMENTS:**

1. If the reviewer comments include a recommendation to cite specific previously published works, please review and evaluate these publications to determine whether they are relevant and should be cited. There is no requirement to cite these works unless the editor has indicated otherwise.

No recommendations to cite specific works were made during this revision.

2. Please review your reference list to ensure that it is complete and correct. If you have cited papers that have been retracted, please include the rationale for doing so in the manuscript text, or remove these references and replace them with relevant current references. Any changes to the reference list should be mentioned in the rebuttal letter that accompanies your revised manuscript. If you need to cite a retracted article, indicate the article’s retracted status in the References list and also include a citation and full reference for the retraction notice.

No changes to the references or citations were made during this revision.

Additional Editor Comments:

Thank you for the revision and for addressing all of the comments. I do have one additional comment regarding the conclusion section. To me, the first three paragraphs of the conclusion would fit better in the discussion section. I understand the PLOS ONE format requirements, but at the end of the day, the conclusion should be a brief and focused summary of the main takeaway, its importance, and the key implication of the study rather than an extended discussion. This will add additional overall clarity to your manuscript.

Thank you for handling our manuscript. We believe the four paragraphs of the conclusion section work best as a unit. Therefore, we have followed the convention of some other articles published in *PLOS One* and have moved these to the first four paragraphs of the revised Discussion section. There is no longer a Conclusions section. We hope that addresses the substance of your concern.

**RESPONSE TO REVIEWERS’ COMMENTS:**

Reviewer's Responses to Questions

Thank you to the two reviewers. We have made changes to the Discussion and Conclusion sections that we believe address the concerns raised by Reviewer 1 and the Editor.

**Comments to the Author**

1. If the authors have adequately addressed your comments raised in a previous round of review and you feel that this manuscript is now acceptable for publication, you may indicate that here to bypass the “Comments to the Author” section, enter your conflict of interest statement in the “Confidential to Editor” section, and submit your "Accept" recommendation.

Reviewer #1: (No Response)

Reviewer #2: All comments have been addressed

2. Is the manuscript technically sound, and do the data support the conclusions?

The manuscript must describe a technically sound piece of scientific research with data that supports the conclusions. Experiments must have been conducted rigorously, with appropriate controls, replication, and sample sizes. The conclusions must be drawn appropriately based on the data presented.

Reviewer #1: Yes

Reviewer #2: Yes

3. Has the statistical analysis been performed appropriately and rigorously?

Reviewer #1: I Don't Know

Reviewer #2: I Don't Know

4. Have the authors made all data underlying the findings in their manuscript fully available?

The [PLOS Data policy [track.editorialmanager.com]](https://urldefense.com/v3/__http:/track.editorialmanager.com/CL0/http:*2F*2Fwww.plosone.org*2Fstatic*2Fpolicies.action*23sharing/1/010f019c97b47560-105801d3-4138-4434-95ab-f1a143028859-000000/8uyF9bJ_qv4bVspOKJCJJpC0YLPNn4svb22wFIA-I9A=252__;JSUlJSU!!PDiH4ENfjr2_Jw!A0zdq8IboNK41SqDMWmKCN1r3hA9udxGMyX9oyj_30suE0q9vrYOs7ZdBzpbySEgy-1m6OERWN1x95y4rMxKMxxLuNQ$) requires authors to make all data underlying the findings described in their manuscript fully available without restriction, with rare exception (please refer to the Data Availability Statement in the manuscript PDF file). The data should be provided as part of the manuscript or its supporting information, or deposited to a public repository. For example, in addition to summary statistics, the data points behind means, medians and variance measures should be available. If there are restrictions on publicly sharing data—e.g. participant privacy or use of data from a third party—those must be specified.

Reviewer #1: Yes

Reviewer #2: Yes

5. Is the manuscript presented in an intelligible fashion and written in standard English?

PLOS ONE does not copyedit accepted manuscripts, so the language in submitted articles must be clear, correct, and unambiguous. Any typographical or grammatical errors should be corrected at revision, so please note any specific errors here.

Reviewer #1: Yes

Reviewer #2: Yes

6. Review Comments to the Author

Please use the space provided to explain your answers to the questions above. You may also include additional comments for the author, including concerns about dual publication, research ethics, or publication ethics. (Please upload your review as an attachment if it exceeds 20,000 characters)

Reviewer #1: I appreciate the authors' work to revise the paper, it is indeed more readable now and with clearly stated findings.

Thank you. We are pleased that you found the paper more readable.

I still think it is overly technical, that some methods and results belong in a supplementary appendix,

We are sorry that we could not agree on where the detailed methods and results descriptions belong, but the editor did not express a view that required revision. Thus, we have made no change in that regard.

and that the conclusions section should be further refined, all as I mentioned in my initial review. I understand the authors disagree with these recommendations, and leave the final decision to the authors and the editor.

The editor has suggested changes to the Conclusion section. We have eliminated the Conclusion section, combining it with the Discussion section. We believe that addresses your concern.

Reviewer #2: I appreciate your efforts and the time you spent, as you have adequately addressed all comments and suggestions.

That’s great. Thank you so much for your effort.
